# Adjuvants in fungicide formulations can be skin sensitizers and cause different types of cell stress responses

**DOI:** 10.1016/j.toxrep.2022.11.004

**Published:** 2022-11-18

**Authors:** Renato Ivan de Ávila, Sofía Carreira Santos, Valentina Siino, Fredrik Levander, Malin Lindstedt, Kathrin S. Zeller

**Affiliations:** Lund University, Department of Immunotechnology, Medicon Village, House 406, 22387 Lund, Sweden

**Keywords:** *in vitro* models, Skin sensitization, Immunotoxicity, Omics approaches, Cocktail effects, Plant protection products

## Abstract

New approaches based on -omics technologies can identify biomarkers and processes regulated in response to xenobiotics, and thus support toxicological risk assessments. This is vital to meet the challenges associated with “cocktail effects”, i.e. combination effects of chemicals present simultaneously in a product, our environment, and/or our body. For plant protection products (PPPs), investigations largely focus on active ingredients such as herbicides and fungicides. In this study, we have analyzed agricultural chemicals, two surfactants (poly(oxy-1,2-ethanediyl), alpha-sulfo-omega-[2,4,6-tris(1-phenylethyl)phenoxy]-, ammonium salt, POL; N,N-dimethylcapramide, NND), and one preservative, 1,2-benzisothiazol-3(2 H)-one (BEN) used as adjuvants in PPPs, and further three fungicide PPPs, Proline EC 250, Shirlan, Folicur Xpert, containing the adjuvants, and other major individual constituents (fluazinam (FLU), prothioconazole (PRO), tebuconazole (TEB)) as well as defined mixtures (“mixes”) thereof using several *in vitro* approaches. All investigated single agricultural chemicals were predicted as skin sensitizers using an *in vitro* transcriptomic assay based on a dendritic cell model. For selected chemicals and mixes, also skin sensitization potency was predicted. The preservative BEN induced significant changes in cytokine secretion and dendritic cell activation marker CD86 expression. The surfactant NND changed cytokine secretion only and the POL only affected CD86 expression. Proteomic analyses revealed unique response profiles for all adjuvants, an oxidative stress pattern response in BEN-treated cells, and differentially abundant proteins associated with cholesterol homeostasis in response to POL. In summary, we find responses to agricultural chemicals and products consistent with the dendritic cell model reacting to chemical exposure with oxidative stress, ER stress, effects on autophagy, and metabolic changes especially related to cholesterol homeostasis. After exposure to certain mixes, novel proteins or transcripts were differentially expressed and these were not detected for any single constituents, supporting the occurrence of cocktail effects. This indicates that all chemicals in a PPP can contribute to the toxicity profile of a PPP, including their skin sensitizing/immunotoxic properties.

## Highlights

• The investigated agricultural chemicals were predicted as skin sensitizers.
• Most chemicals induced unique cellular responses.
• Complex response patterns occur in mixes, and cocktail effects may emerge.
• Adjuvants can contribute to fungicide formulation immunotoxicity.

## Graphical Abstract

**Figure:**
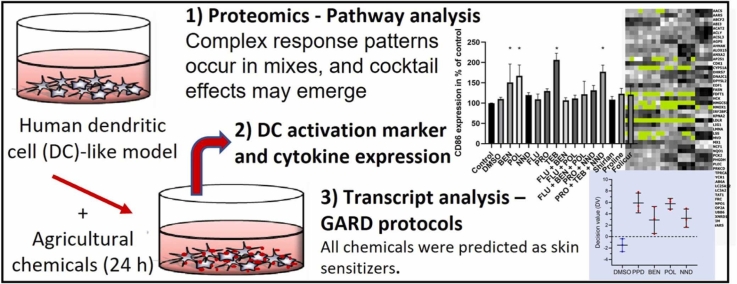

## 1 Introduction

New approach methods (NAMs) based on -omics technologies can be used to identify biomarkers and processes regulated in response to xenobiotic exposure, and thus to support toxicological risk assessments. Traditionally, toxicological assessments have focused on one substance at a time, however, more attention is now paid to the so-called “cocktail effects”, i.e. combination effects of several chemicals present simultaneously in a product, in our environment, and/or in our body. These effects can be additive, synergistic, or antagonistic [1], [2] and new approaches are needed to meet the challenges associated with cocktail effects in risk assessment.

Plant protection products, PPPs, are mixtures of several chemicals, divided into active principle, the pesticide as such, and “inert ingredients”, also called adjuvants. The latter are, despite their name, not inert and are added to enhance product performance and stability, e.g. surfactants and preservatives.

Pesticides need to undergo toxicity testing before being approved for use. Active ingredients are regulated on the European level, while each member state decides about the authorization of the PPPs in the respective country [3]. Exposure to pesticides can occur for instance through contact in the occupational setting, or household usage of pesticides and residues in products. There is a concern that they may cause adverse health effects, among others, immunotoxicity including skin sensitization leading to allergic contact dermatitis (ACD) [4], [5].

In the present study, we use a well-characterized myeloid cell model, resembling dendritic cells (DCs), the sentinels of our immune system, to investigate skin sensitizing and immunotoxic properties of a selection of fungicides, PPPs, and adjuvants used in agriculture, both alone and in combination. DCs form a crucial bridge between innate and adaptive immunity and this important role is also reflected by their central place in the skin sensitization adverse outcome pathway [6]. The myeloid cell line we used as a DC model can display phenotypes and expression profiles similar to those of immature and mature DCs upon stimulation [7], [8]. It can present antigens through MHC class I and II as well as CD1d and induce speciﬁc T-cell proliferation [9]. This cell line has also been used to develop an *in vitro* test system for the prediction of skin sensitization using a transcriptomic approach [10], [11], [12], now further developed as GARD® technology [13]. GARD®skin has recently been approved by the Organisation for Economic Co-operation and Development (OECD) as part of Test Guideline 442E “*In vitro* Skin Sensitization” [14].

Skin sensitization and its key events [15] are well understood compared to other immunotoxicities such as autoimmune reactions and some other hypersensitivities but more knowledge about involved molecular mechanisms is needed to further advance predictive *in vitro* assays and develop mechanism-based therapy [16].

Inflammation needs to be triggered by molecular cues signaling “danger”. Without proper danger signals, DCs will not be able to activate allergen-specific T cells and thus, no adaptive immune response, i.e. sensitization, will occur [17], [18]. The danger signals can derive from irritants such as cytotoxic properties of the sensitizer itself, or be delivered through other chemicals present simultaneously, such as described for the detergent sodium dodecyl sulfate (SDS) [19], [20]. We, therefore, hypothesized that adjuvants, including surfactants, used in PPPs could be of major importance for the toxicity profile of the complete PPP, despite the regulatory focus on the active principle alone. Cocktail effects in the context of skin sensitization in response to agricultural chemicals have to our knowledge not been addressed.

With thousands of chemicals identified as skin sensitizers, covering a wide range of structures and reactivities, it is naturally challenging to delineate universally applicable pathways triggered by sensitizers, especially if the response to mixtures of chemicals is to be understood. However, several *in vitro* assays based on different model systems provide prediction accuracies that are at least competitive with the traditional animal models for skin sensitizer identification such as the Local Lymph Node Assay (LLNA) [21], including GARD® and others [13], [22].

In the first part of this study, we focused on two agricultural surfactants and one preservative. In the second part, we analyzed three commercially available fungicide PPPs, which contain the adjuvants analyzed in the first part and the major individual constituents of the PPPs. We predicted the skin sensitization capacity of these chemicals, including some defined mixtures (“mixes”) and the PPPs, and the skin sensitization potency for selected chemicals and mixes based on customized protocols adapted from the GARD® transcriptomic assay [12], [23]. These classifications were then compared to existing human and animal data. We further evaluated the expression of CD86, and for the three adjuvants, we also profiled cytokine expression using Luminex® technology. Furthermore, changes induced at the proteome level and selected transcripts after exposure to the respective chemicals and mixes were investigated to map molecular and cellular responses related to skin sensitization and immunotoxicity and used to perform pathway analysis.

## 2 Materials and methods

### 2.1 Materials

The surfactant poly(oxy-1,2-ethanediyl), alpha-sulfo-omega-[2,4,6-tris(1-phenylethyl)phenoxy]-, ammonium salt was obtained from Alfa Chemistry (Stony Brook, NY, USA) with an average molecular weight of 1225 g/mol. This preparation also contained 1–3% Tristyrylphenol ethoxylate. The fungicide formulations Folicur Xpert, Proline EC 250 and Shirlan were acquired from Svensk Växtskydd (Stockholm, Sweden) via the Rural Economy and Agricultural Society (Hushållningssällskap, Bjärred, Sweden). Other chemicals, including all remaining agricultural chemicals, were acquired from Sigma-Aldrich (St. Louis, MO, USA) if not indicated otherwise.

HyClone™ minimum essential medium α-modification with L-glutamine, ribo- and deoxyribonucleosides (MEM-α), fetal bovine serum (FBS), Trypan Blue, and TRizol reagent were purchased from Thermo Fisher Scientific (Waltham, MA, USA). BSA Cohn fraction V was obtained from Saveen&Werner (Limhamn, Sweden). Recombinant human granulocyte-macrophage colony-stimulating factor (rhGM-CSF) was purchased from PeproTech (Rocky Hill, NJ, USA). Propidium Iodide (PI), FITC-conjugated anti-human [isotype control anti-IgG1 (MOPC-21), CD86 (FUN-1), HLA-DR (L243) and CD34 (581)] antibodies and PE-conjugated anti-human (isotype control anti-IgG1 (MOPC-21), CD54 (HA58) and CD80 (L307)) antibodies were purchased from BD Biosciences (San Jose, CA, USA), whereas FITC-conjugated anti-human CD1a (NA1/34) and PE-conjugated anti-human CD14 (TÜK4) were obtained from Dako (Santa Clara, CA, USA). Direct-zol RNA MiniPrep column purification kit was purchased from Zymo Research (Irvine, CA, USA), whereas reagents to perform GARD™ skin assay were acquired from NanoString Technologies (Seattle, WA, USA).

#### 2.1.1 Cell culture

MUTZ-3 cells (DSMZ, Braunschweig, Germany), were cultured in MEM-α medium, supplemented with 20% FBS (v/v) and rhGM-CSF (40 ng/mL), and maintained in a cell incubator under controlled conditions (humidified atmosphere at 37ºC and 5% CO_2_ in air). Experiments were carried out using different batches of cells with satisfactory cell viability (>85%), a parameter that was estimated using a LUNA™ automated cell counter (Logos Biosystems, Annandale, VA, USA) using Trypan Blue. Before every transcriptomic test, a cell phenotypic quality control was carried out following previously published protocols [13], [24]. In brief, the expression of the following biomarkers was investigated using BD FACSCanto II flow cytometer (BD Biosciences, San Jose, CA, USA): CD86, HLA-DR, CD34, CD1a, CD56, CD80, and CD14. In addition, PI (1 µg/mL) staining was used to assess cell viability.

#### 2.1.2 Test materials set

The three commercial fungicide formulations or PPPs tested in this study were chosen due to their frequent use in Sweden (Table 1, Table 2). Then, their active ingredients and adjuvants were acquired depending on commercial availability to investigate their toxicological effects when tested alone or in different combinations thereof (i.e. active ingredient + adjuvant). These defined mixtures mimicking a formulation (here also called mixes) were prepared based on the concentration ratios of these chemicals found in the fungicide formulation according to the supplier and the requirement to target relative viability of 90%. If a range was indicated, the average concentration was used for calculation (Table 2). The fungicide formulations were dissolved in medium, whereas other test materials were solubilized in dimethyl sulfoxide (DMSO) and then diluted in medium with a maximal DMSO concentration of 0.01% v/v.

**Table 1:** Overview of the used chemicals, used input concentrations and existing skin sensitization data /classifications.

	Abbreviation	Classification			Input concentration
		CLP category	Animal data	Human evidence	
Reference controls (CAS no.)					
Dimethyl sulfoxide (67–68–5)	DMSO	No cat.	Negative	Negative	0.1% (v/v)
*p*-Phenylenediamine (106–50–3)	PPD	Skin Sens. 1A	Positive	Positive	75 µM
Fungicide active ingredients (CAS no.)					
Prothioconazole (178928–70–6)	PRO	No cat.	Negative^a,c^		115 µM
Tebuconazole (107534–96–3)	TEB	No cat.	Negative^b,d^		125 µM
Fluazinam (79622–59–6)	FLU	Skin Sens. 1A	Positive^b^/Negative^c^	Positive^f^	3 µM
Fungicide adjuvants (CAS no.)					
Poly(oxy-1,2-ethanediyl), alpha-sulfo-omega-[2,4,6-tris(1-phenylethyl)phenoxy]-, ammonium salt (119432–41–6)	POL	No cat.			500 µM
N,N-Dimethylcapramide (14433–76–2)	NND	No cat.			220 µM
1,2-Benzisothiazol-3(2 H)-one (2634–33–5)	BEN	Skin Sens. 1	Positive^e,c^	Positive^e^	6.5 µM
Defined mixtures					
FLU (3 µM) + BEN (0.0132 µM)	Mix 4				3.01 µM
FLU (3 µM) + POL (0.091 µM)	Mix 5				3.09 µM
FLU (3 µM) + BEN (0.0132 µM) + POL (0.0914 µM)	Mix 6				3.10 µM
PRO (86.25 µM) + NND (119.18 µM)	Mix 7				205.43 µM
PRO (28.6 µM) + TEB (63.96 µM) + NND (121 µM)	Mix 11				213.56 µM
Commercial fungicide formulations (KEMI registration no.)					
Proline EC 250 (4688)	Proline	No cat.			58 µg/mL
Shirlan (3957)	Shirlan	Skin Sens. 1B		Positive^f^	12 µg/mL
Folicur Xpert (5413)	Folicur	Skin Sens. 1			40 µg/mL

Abbreviations: CLP – Harmonized classification, labelling and packaging**;** KEMI –*Kemikalieinspektionen* (The Swedish Chemicals Agency).

Human evidence regarding skin sensitization hazard according to ^f^Ginkel and Sabapathy [46].

^a^Classification based on the murine local lymph node assay (LLNA) according to the European Food Safety Authority (EFSA).

^b^Classification based on the guinea-pig maximization test/Buehler test according to the EFSA.

^c^Classification based on the guinea-pig maximization test/Buehler test according to the United States Environmental Protection Agency (US EPA).

^d^Animal classification according to US EPA data (*in vivo* method not specified).

^e^Classification based on the murine local lymph node assay (LLNA), the guinea-pig maximization test/Buehler test, or human data according to Scientific Committee on Consumer Safety (SCCS).

**Table 2:** Commercial fungicide formulations composition tested. Ingredients and formulations highlighted in bold are classified as skin sensitizers according to Harmonized Classification, Labelling and Packaging (CLP) system.

	Formulation type	Manufacturer	Composition (%, w/w) stated by the manufacturers^a^	
			Active ingredients	Adjuvants
Proline EC 250	EC	Bayer	Prothioconazole: 25	N,N-Dimethylcapramide: > 20
Shirlan	SC	ISK Biosciences	Fluazinam: 25–50	1,2-Benzisothiazol-3(2 H)-one: < 0.05; methenamine: 0.5–1; poly(oxy-1,2-ethanediyl), alpha-sulfo-omega-[2,4,6-tris(1-phenylethyl)phenoxy]-, ammonium salt: 1–5; Alkylated naphthalene sulfonate sodium salt: 3.5–5; fumaric acid: 1–1.5
Folicur Xpert	EC	Bayer	Prothioconazol: 8.15; tebuconazole: 16.3	2-[2-(1-chlorocyclopropyl)− 2-hydroxy-3-phenylpropyl]− 2,4-dihydro-1,2,4-triazole-3-thione: > 0.1-< 1; N,N-Dimethylcapramide: > 20

Abbreviations: EC - Emulsion concentrate; SC - Suspension concentrate.

a Some ingredients are confidential. Thus, suppliers only mentioned in the material safety data sheet those that are mandatory according to the current legislation and classification

#### 2.1.3 Cytotoxicity analysis

Cells were exposed to the test materials according to published protocols [13], [24], [25] to determine the input non-cytotoxic concentrations for the tested chemicals and mixtures, i.e. chemical concentrations, which resulted in 90% relative viability when compared to unstimulated cells (RV_90_).

In brief, cells (2 ×10^5^ cells/mL) were exposed to different concentrations of test materials, previously diluted in suitable vehicles as described above. After 24 h, cells were stained with PI (1 µg/mL) followed by flow cytometry analysis to evaluate cell viability. The RV_90_ values for each test material were then obtained and used in further analyses as input concentrations (Table 1). For non-cytotoxic pure substances, an input concentration of 500 µM was set up.

#### 2.1.4 CD86 expression analysis

After 24 h of exposure, cells were washed twice with cold wash buffer (PBS containing 0.5% (w/v) BSA Cohn fraction V and centrifuged at 1200 rpm, 5 min, 4 °C. They were then stained with a solution containing PI, FITC-conjugated anti-human CD86, or isotype control anti-IgG1 followed by incubation at 2–8 °C for 10 min. Cells were then washed, resuspended in 200 µL wash buffer, and analyzed by BD FACSCanto II flow cytometer recording 10,000 events. PI+ cells representing dead cells were excluded from the CD86 expression analysis.

#### 2.1.5 Transcriptomic test to predict skin sensitization

This step was performed according to published guidelines for the GARD assay and following GARD® technology protocols [13], [24], [25]. In brief, three different batches of cells (2 × 10^5^ cells/mL) were exposed to the test materials for 24 h. Right after, cell samples were collected to perform PI staining and then the cell viability was analyzed. Samples passing with a relative viability between 84.5% and 95.4% were lysed in TRizol and stored at − 20 °C until the total RNA extraction procedure. This step was then performed using the Direct-zol RNA MiniPrep column purification kit (Zymo Research, Irvine, USA) and integrity and concentration of total RNA samples were evaluated using Agilent Bioanalyzer 2100 (Agilent Technologies, Santa Clara, CA, USA), following manufacturer´s instructions. RNA samples were then transferred to Senzagen AB, Lund, Sweden, where the remaining steps for GARD®skin and GARD®potency were performed. For GARD gene signature analyses the NanoString GEN2 nCounter Analysis System (NanoString Technologies, Seattle, USA) was used and analysis was performed according to protocols provided by the supplier. In short, RNA samples were thawed on ice and subjected to quality control (Agilent 2100 Bioanalyzer, Agilent Technologies, Santa Clara, California). RNA samples were hybridized to the assay-specific probe pairs and analyzed using recommended kits and reagents. Test materials were classified as skin sensitizers when the mean of the support vector machine decision values obtained for triplicate samples was > 0. Some of the skin sensitizers were further predicted into 1A and 1B potency classes [26]. The potency predictions are based on the same physical samples and processing as for the GARD®skin based assay, with the difference that RNA expression was analyzed for a different biomarker signature consisting of 52 transcripts and processed with another algorithm that also takes the input concentration into account [23], [26]. Senzagen AB did not know about the identity of the substances used to expose the cell model at the time of analysis.

#### 2.1.6 Transcript analysis

Quantified transcriptional levels obtained for the transcripts evaluated in GARD®skin were normalized using the counts per total counts (CPTC) method [24] scaling the expression levels for the respective sample by the total number of acquired counts for that sample. Following CPTC, the data were rescaled by the mean total number of acquired counts for all samples in the dataset, and then log-transformed. Qlucore Omics Explorer 3.7 (Qlucore AB, Lund, Sweden) was used to identify differentially abundant transcripts (false discovery rate FDR≤0.05 or FDR≤0.01 as indicated) and to visualize the RNA expression data as heat maps.

#### 2.1.7 Multiplex cytokine analysis

After centrifugation at 1200 rpm, 5 min, 4 °C, the supernatants including the test materials were separated from the cell pellets, which are used for proteomics analysis.

The following cytokines were profiled using a customized Premixed Human Magnetic Luminex® assay kit from R&D systems (Biotechne, Minneapolis, USA): IL-1α/IL-1F1, IL-1β/IL-1F2, IFN-γ, IL-8/CXCL8, IL-6, IL-10, IL-15, IL12/IL23 p40, IL18/IL-1F4, TNF-α. Only IL-8 was measurable above detection limits. Samples were measured in technical duplicates according to the manufacturer’s recommendation using a Bio-Plex 200 (Bio-Rad Laboratories, California, USA). The coefficient of variation was always below 10%.

### 2.2 Proteomics

#### 2.2.1 Protein and peptide extraction for mass spectrometry

Cell pellets, corresponding to approx. 1 million cells, were dissolved in 200 µL lysis buffer (5% SDS and 50 mM Tris, pH=7.55) and homogenized using probe sonication on ice. Debris was removed by centrifugation and proteins were quantified with the Pierce BCA protein assay kit (Thermo Fisher Scientific, Germany). 50 µg of proteins were used for hydrophilic interaction liquid chromatography (HILIC, ReSyn Biosciences, South Africa) clean-up and automated protein on-bead digestion with trypsin using KingFisher Flex (Thermo Fisher Scientific, Germany) in a 96-well format.

Peptides were recovered from the plate and dried in a Speedvac (Thermo Fisher Scientific, Germany) prior to C18 desalting using BioPureSPN Mini, PROTO 300 C18 (The Nest Group, Inc., MA, USA). Cleaned peptides were dried in the Speedvac and stored at − 20 °C before quantification and injection into the mass spectrometer.

#### 2.2.2 Mass spectrometry analysis

Cleaned peptide digests were quantified using the NanoDrop and approximnately 300 ng were injected on an EASY-nano LC system 1200 (Thermo Fisher Scientific, Germany) and separated using a 60 min gradient on a 15 cm fused silica capillary with Pico Tip emitter (New Objective) packed with 1.9 µm C18 ReproSil-Pur C18 material. The LC was coupled with a QExactive HF-X mass spectrometer (Thermo Fisher Scientific, Germany) operating in positive ion mode with data-dependent acquisition (DDA). A top-20 method was used for selection of peptide ions for higher energy collision-induced dissociation fragmentation (normalized collision energy: 40 V), with target values of 3 × 10^6^ and 1 × 10^5^ ions for MS and MS/MS, respectively.

#### 2.2.3 Mass spectrometry data processing

The generated RAW files were processed using MaxQuant (www.maxquant.org, version 1.6.10.43). Files were searched against the uniport human proteome database as of 4th June 2020 using the following parameters: carbamidomethylation of cysteines as fixed modification and oxidation of methionine and protein N-terminal acetylation as variable modifications. Default parameters were used, including precursor mass error of 4.5 ppm and monoisotopic fragments mass error of 0.02 Da and protein filtering at FDR ≤ 0.01.

The protein intensity values from the resulting protein groups file were normalized using NormalyzerDE [27]. Cyclic Loess normalization was chosen as the best normalization method based on the metrics in the report since it adjusts for systematic differences in abundance between samples at different abundance levels [28]. The normalized protein list (log2 transformed) was exported and used for further data analysis.

Qlucore Omics Explorer 3.7 (Qlucore AB, Lund, Sweden) was used to identify differentially abundant proteins (false discovery rate FDR≤0.05 or FDR≤0.01 as indicated) and to visualize the protein abundance data as heat maps and Principal Component Analysis (PCA) plots after eliminating the factor “main stimulation”. The mass spectrometry proteomics data have been deposited to the ProteomeXchange Consortium via the PRIDE [29] partner repository with the dataset identifier PXD034624, project name: Myeloid cell responses to fungicides, surfactants and fungicide formulations.

#### 2.2.4 Key advisor pathway (KPA) analysis and other statistical analysis

Data are expressed as mean or mean ± SD of three independent assays. For CD86 expression data, intergroup variation was evaluated by one-way Analysis of Variance (ANOVA) followed by Dunnett´s multiple comparisons test by a statistical significance at p < 0.05 using GraphPad Prism (GraphPad, San Diego, CA, USA). IL-8 expression was analyzed using Microsoft Excel and Student’s t-test (p < 0.05) and visualized in GraphPad Prism.

To identify common and unique transcripts, pathways, and proteins, the web service interactivenn was used [30].

For KPA analysis version 17.4 was used. Input files (proteomics) were generated based on two-group comparisons between respective treatments again both untreated and vehicle control with a cut-off of p ≤ 0.05. The resulting list containing associated gene symbols, p-value, and fold change was uploaded into the KPA tool and analyzed under standard settings by applying Causal Reasoning Analysis.

## 3 Results

### 3.1 All adjuvants are predicted to be skin sensitizers

**Fig. 1:**
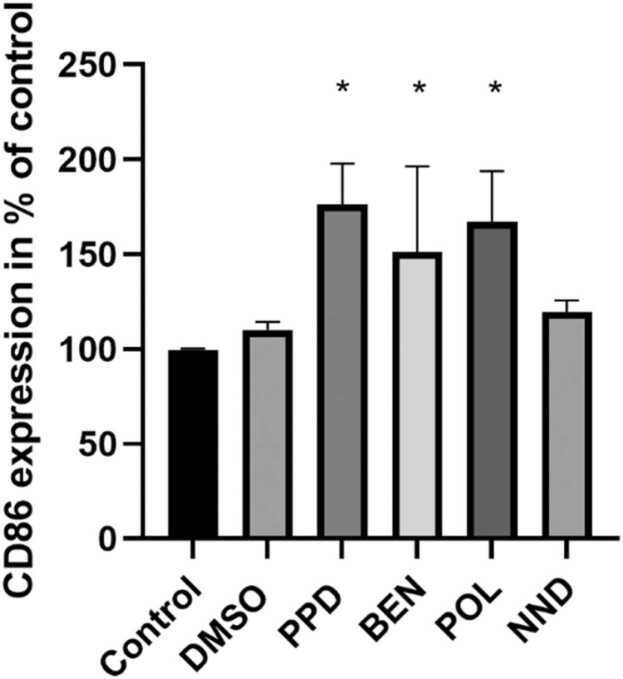
CD86 expression investigated by flow cytometry after 24 h of stimulation with indicated chemicals or controls. The mean % of positive cells in treated sample/mean % of positive cells in untreated sample is shown (n = 3). Cells were treated with 75 µM PPD, 6.5 µM BEN, 500 µM POL, and 220 µM NND. * p < 0.05 compared to control (untreated). Error bars represent standard deviation.

Benzisothiazol-3(2 H)-one (BEN), poly(oxy-1,2-ethanediyl), alpha-sulfo-omega-[2,4,6-tris(1-phenylethyl)phenoxy]-, ammonium salt (POL) and (N,N-dimethylcapramide) NND input concentrations resulting in 90% relative cell viability of the DC model were determined (Table 1) and the CD86 expression was assessed by flow cytometry after 24 h incubation with the respective chemical. POL did not show any cytotoxicity at the maximum tested concentration of 500 µM. The known sensitizer para-phenylenediamine (PPD, positive control), the cytotoxic preservative BEN and the surfactant POL induced a significant increase in the percentage of positive cells for the activation marker CD86 in comparison to control cells, while the surfactant NND did not (Fig. 1).

**Fig. 2:**
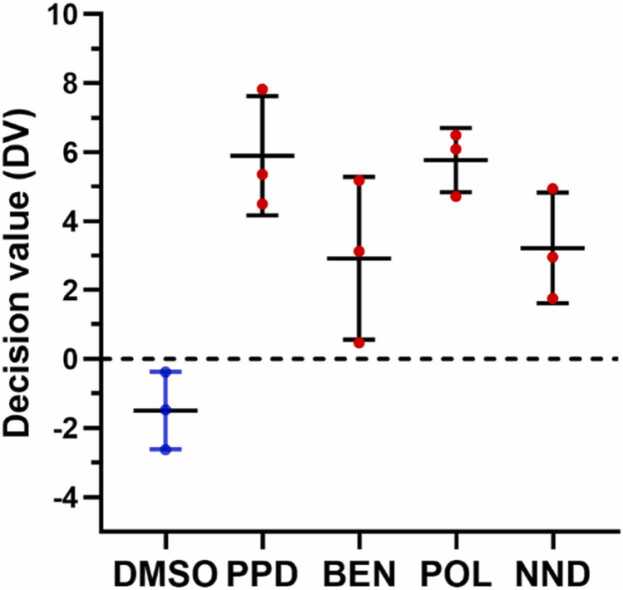
Decision values (DV) based on GARD®skin predictions. Chemicals were classified as skin sensitizers (red symbols) when the mean of the support vector machine DV for triplicate samples was > 0. Cells were treated with 75 µM PPD, 6.5 µM BEN, 500 µM POL, and 220 µM NND. Mean with standard deviation is shown.

All three investigated adjuvants were predicted as skin sensitizers (Fig. 2) based on the GARD® biomarker signature comprising 196 transcripts and additional potency classifications based on a biomarker signature with 51 transcripts resulted in category 1A (strong sensitizer) for BEN and POL and category 1B for NND.

### 3.2 Differentially expressed proteins and pathway analysis indicate distinct response patterns following exposure to each adjuvant

**Fig. 3:**
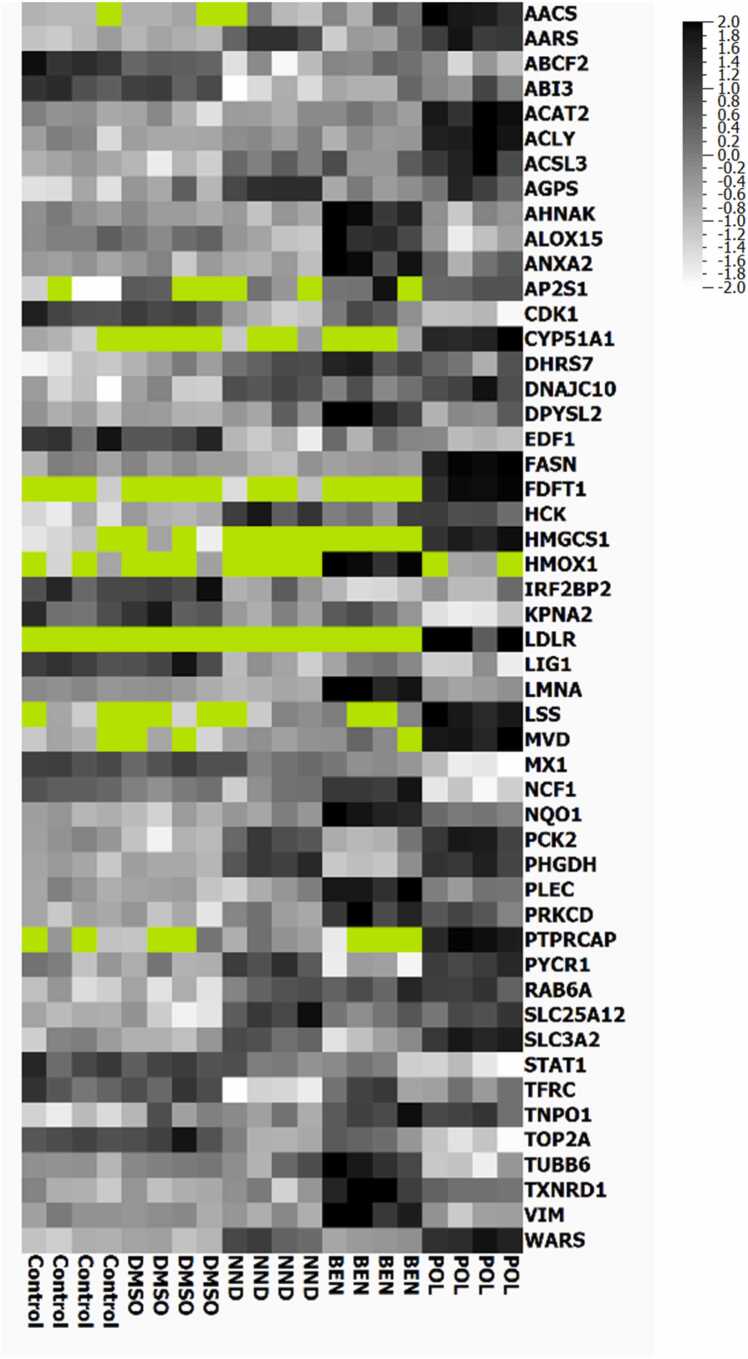
Heatmap illustrating the multigroup comparison (FDR < 0.05, 50 differentially expressed proteins) between the proteomes of cells exposed to adjuvants and indicated controls. Cells were treated with 6.5 µM BEN, 500 µM POL, and 220 µM NND. Dark color indicates high expression levels, white low. Missing values are shown in light green, normalized to mean= 0, variance= 1.

**Fig. 4:**
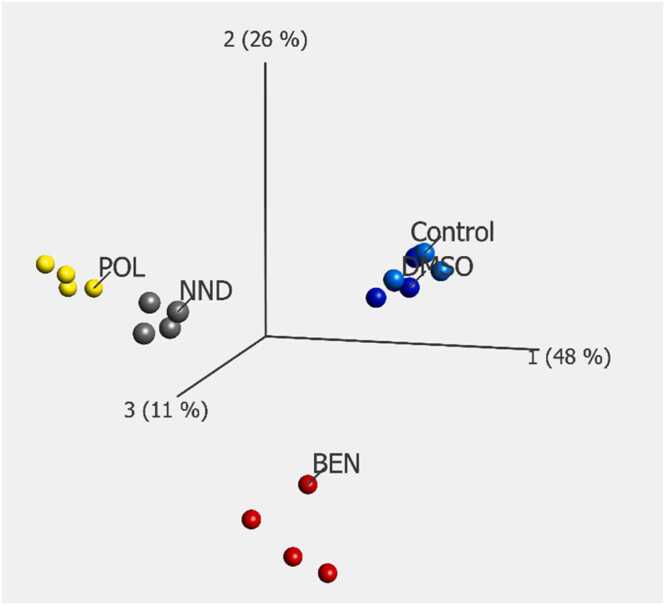
Principal component analysis visualizing the multigroup comparison (FDR < 0.05) between the proteomes of cells exposed to adjuvants and indicated controls (Control and vehicle blue, NND grey, POL yellow, BEN red. 50 differentially abundant proteins). Cells were treated with 75 µM PPD, 6.5 µM BEN, 500 µM POL, and 220 µM NND.

To investigate protein-level changes introduced by the different chemicals, we employed an LC-MS/MS proteomics methodology that allowed for the quantification of more than 5000 protein groups. A multigroup comparison (FDR < 0.05) based on the protein profiles detected in BEN-, NND-, POL-treated, and control cells identified 50 proteins with differential abundance (visualized in the heatmap, Fig. 3), among them the proteins Tryptophanyl-tRNA synthetase (WARS), Fatty acid synthase (FASN), NAD(P)H dehydrogenase [quinone] 1 (NQO1), D-3-phosphoglycerate dehydrogenase (PHGDH) and Heme oxygenase 1 (HMOX1) were highly significant. Individual comparisons of each adjuvant to controls (FDR < 0.05) revealed no common differentially abundant proteins (Suppl. Table 1). In contrast, the surfactants POL and NND shared 4 of 27 and 15, respectively, proteins significantly different from controls, which is also mirrored by their proximity in the PCA plot (Fig. 4). Unique responses were dominated by an oxidative stress pattern response in BEN-treated cells and differentially abundant proteins associated with cholesterol synthesis and homeostasis in POL-treated cells. The top 5 proteins with increased abundance compared to the controls were Lanosterol 14-alpha demethylase (CYP51A1), Lanosterol synthase (LSS), Squalene synthase (FDFT1), Sterol O-acyltransferase 2 (ACAT2), and Diphosphomevalonate decarboxylase (MVD), with fold changes between 5.3 and 2.6 times. The proteins which showed the lowest abundance levels compared to the controls were Interferon-induced GTP-binding protein (MX1), DNA topoisomerase 2-alpha (TOP2A), and Signal transducer and activator of transcription 1-alpha/beta (STAT1) (Suppl. Table 1). Proteins with the highest fold changes in response to NND were associated with a wide variety of processes, including metabolism, kinase activity regulation, and the endoplasmic reticulum-associated degradation (ERAD) pathway.

Key pathway advisor analysis of the proteome indicated regulation of several directly immune system-associated pathways activated in cells exposed to BEN (18/87) and NND (2/40), but none for cells exposed to POL (0/6) (Table 3). Hits for BEN-treated cells comprised several pathways associated with NF-ΚB signaling and “Oxidative stress - ROS-induced cellular signaling”, and none of these were predicted for cells exposed to NND or POL. Prediction of pathway regulation for POL-treated cells instead was dominated by pathways linked to fatty acid, lipid, and cholesterol metabolism (Table 3).

**Table 3:** Key Pathway Advisor pathway predictions for adjuvants. Analysis based on the input of differentially expressed proteins (p = 0.05) induced by exposure of the DC model to the indicated adjuvants against controls. In brackets: total number of pathways predicted.

Pathway associated with	BEN (87)	NND (40)	POL (6)
Fatty acid, lipid, and cholesterol metabolism/homeostasis	–	–	5
Other metabolism	1	2	–
ER stress	–	–	1
Chemotaxis/cell adhesion/cytoskeletal re-arrangements	4	–	–
Oxidative stress: ROS-induced cellular signaling	1	–	–
DNA damage	2	–	–
NF-ΚB-associated signaling	5	–	–
Specific signal transduction	9	5	–
Number of cytokine and immune response-associated pathways	18	2	0

### 3.3 Transcript data in line with differentially expressed proteins

To identify differentially expressed transcripts in response to BEN, NND, and POL, we analyzed the 196 transcripts evaluated in the context of the GARD prediction signature (GPS) [12], [25]. Despite differences in the approaches, one using pre-defined transcripts chosen to predict characteristics correlating with skin sensitizing capacity, and one global approach looking at all detectable proteins/peptides, the overall picture for BEN-and POL-treated cells pointed out similar trends: in BEN-treated cells, molecules associated with oxidative stress were also on transcript level among the differentially abundant ones with high fold change. In POL-treated cells, several differentially abundant transcripts were associated with fatty acid/cholesterol synthesis and homeostasis (e.g. CYP51A1, DHCR7, DHCR24, SREBF2, FDXR), as also seen at the protein level. Some cholesterol synthesis/homeostasis-related transcripts were differentially abundant also in cells exposed to NND, not appearing in the corresponding differentially abundance protein list (Suppl. Table 1). In NND-treated cells, no common transcripts and protein IDs were identified.

Commonly differentially expressed transcripts and proteins are summarized in Table 4 with respective fold changes, correlating in both direction and magnitude.

**Table 4:** Commonly differentially expressed transcripts and proteins in response to adjuvants.

Cells treated with	Gene name for transcript/protein differentially expressed	Fold change protein	Fold change transcript
BEN	NQO1 TXNRD1	4.9 1.5	3.4 1.6
NND	–		
POL	CYP51A1 FASN ACLY	5.3 1.6 1.5	4.7 1.8 1.4

### 3.4 BEN and NND influence IL-8 secretion

**Fig. 5:**
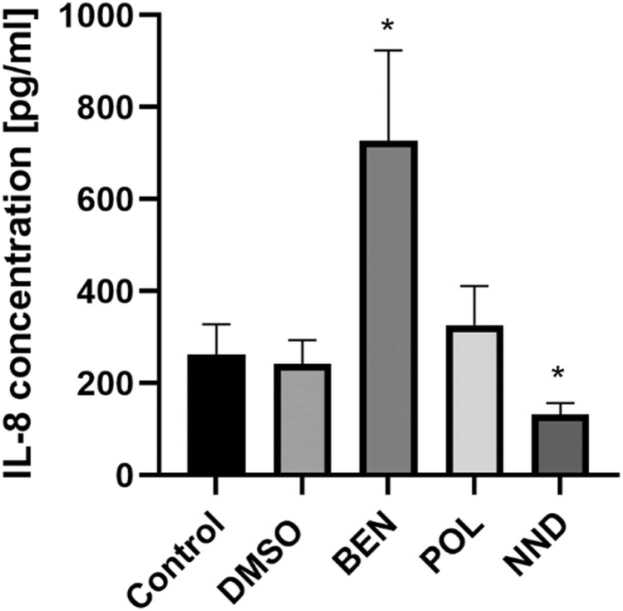
IL-8 concentrations in the supernatant after treatment with respective adjuvant for 24 h. n = 4 (2 biological replicates) for control and DMSO, n = 8 (4 biological replicates) for BEN, POL, and NND. Cells were treated with 6.5 µM BEN, 500 µM POL, and 220 µM NND. Mean values and SD are presented, p < 0.05.

We also analyzed the supernatants harvested simultaneously with RNA and protein using a multiplex cytokine assay. Of 10 investigated cytokines, only IL-8 was secreted above detection level. NND exposure led to a decrease in IL-8 while BEN treatment induced an increase in IL-8 concentration in the cell supernatant, and no changes were detected after exposure to POL (Fig. 5).

### 3.5 Investigation of 3 commercial fungicide formulations

We also investigated 3 fungicide formulations (Proline, Folicur Xpert, and Shirlan), which contained at least one of the previously described adjuvants (Table 1, Table 2) and associated single ingredients (Prothioconazole (PRO), Tebuconazole (TEB), Fluazinam (FLU)) and mixes to map potential cocktail effects. The formulations and associated single chemicals and mixes were:

(1) PRO-NND-Mix 7 – Proline
(2) PRO-TEB-NND-Mix 11 – Folicur Xpert
(3) FLU-BEN-POL-Mix 4-Mix 5-Mix 6 - Shirlan

**Fig. 6:**
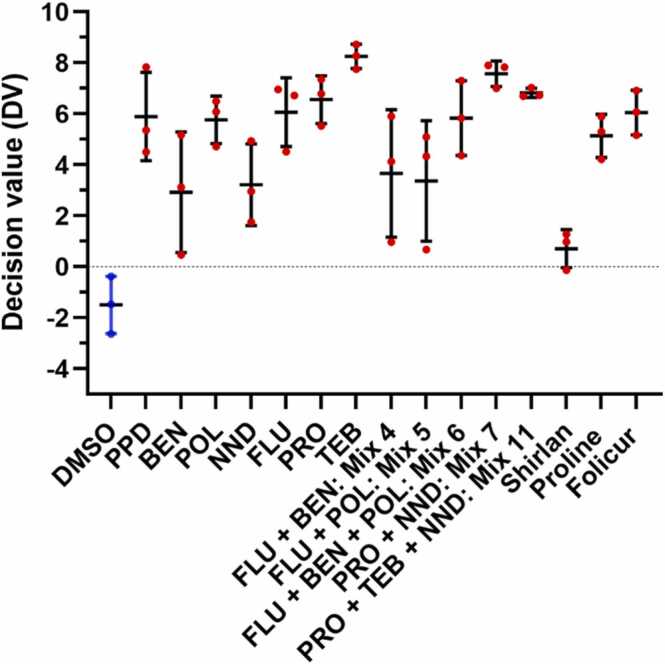
Decision values (DV) based on GARD®skin predictions. Chemicals were classified as skin sensitizers (red symbols) when the mean of the support vector machine DV for triplicate samples was > 0. To simplify comparisons, BEN (6.5 µM), POL (500 µM), and NND (220 µM) values have been included. FLU 3 µM, PRO 115 µM, TEB 125 µM, Mix 4: FLU+BEN (3 µM + 0.0132 µM); Mix 5: FLU+POL (3 µM + 0.091 µM); Mix 6: FLU+BEN+POL (3 µM + 0.0132 µM+0.091 µM); Mix 7: PRO+NND (86 µM + 119 µM); Mix 11: PRO+TEB+NND (28.6 µM + 64 µM+121 µM), Shirlan 12 µg/mL; Proline 58 µg/mL; Folicur 40 µg/mL. Mean with standard deviation is shown.

Predictions, based on GARD® technology, classified all investigated single ingredients, mixes, and formulations as skin sensitizing (Fig. 6). Further, FLU, PRO, and TEB were predicted to be category 1 A sensitizers, as were Mixes 4, 5, and 6 mimicking different mixtures related to Shirlan with adjuvants BEN and POL (themselves predicted as category 1 A). Mix 7 and Mix 11, mimicking Proline and Folicur Xpert, respectively, were predicted as category 1B sensitizers. Mix 7 and Mix 11 contain NND, itself predicted as category 1B. We chose to not apply potency predictions to the formulations.

**Fig. 7:**
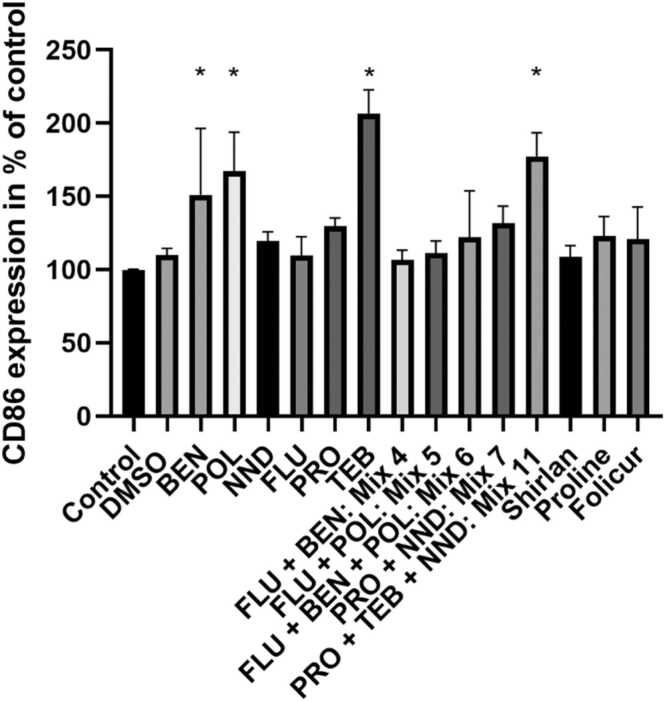
CD86 expression investigated by flow cytometry after 24 h of stimulation with indicated chemicals or controls. The mean % of positive cells in treated sample/mean % of positive cells in untreated sample is shown is shown (n = 3). *p < 0.05 compared to control (untreated). Error bars represent standard deviation. To simplify comparisons, BEN (6.5 µM), POL (500 µM), and NND (220 µM) values have been included. FLU 3 µM, PRO 115 µM, TEB 125 µM, Mix 4: FLU+BEN (3 µM + 0.0132 µM); Mix 5: FLU+POL (3 µM + 0.091 µM); Mix 6: FLU+BEN+POL (3 µM + 0.0132 µM+0.091 µM); Mix 7: PRO+NND (86 µM + 119 µM); Mix 11: PRO+TEB+NND (28.6 µM + 64 µM+121 µM); Shirlan 12 µg/mL; Proline 58 µg/mL; Folicur 40 µg/mL.

CD86 expression of the DC model in response to chemical exposure was assessed and the resulting percentages of positive cells compared to control, normalized to 100%, are presented in Fig. 7. Significantly increased percentages of CD86^+^ cells were found after TEB treatment and in response to Mix 11 containing TEB.

In Table 1, previously published classifications based on both animal and human data are summarized. The formulations Shirlan and Folicur Xpert are classified as CLP category 1B and 1, respectively. BEN is considered category 1 and FLU category 1A. The remaining substances belong to “no category” with regard to skin sensitization.

### 3.6 Differentially expressed transcripts and/or proteins for case studies and KPA pathway analysis for PRO, NND, Mix 7, and Proline

PRO induced most differentially expressed proteins of all active ingredients, i.e. fungicides, 70 proteins, in the here described data set. The formulation Proline, based on the chemicals PRO and NND, induced changes in the abundance of 73 proteins (all based on an FDR ≤ 0.05), 39 of which did not appear as differentially abundant in either PRO or NND alone. Examples of newly appearing proteins are the Nuclear factor NF-kappa-B p105 subunit (NFKB1) and Leukocyte elastase inhibitor (SERPINB1), involved in cellular homeostasis and inflammatory response [31]. Others are associated with metabolic processes, innate immunity, and redox balance (Suppl. Table 1).

Using Key pathway advisor (KPA) analysis, only 2 pathways were predicted to be regulated in response to Proline, 16 pathways in response to PRO alone. An overview of PRO-induced pathways is presented in Table 5; the 2 pathways predicted for Proline are associated with glucose and/or fatty acid metabolism and with cytoskeletal reorganization and endosomal-autophagic pathways.

**Table 5:** Key Pathway Advisor pathway predictions for agricultural chemicals and PPPs. Analysis based on the input of differentially expressed proteins (p = 0.05) induced by exposure of the DC model to the indicated substances against controls. In brackets: total number of pathways predicted. Proline: A closer look at the pathways could place one of them in category metabolism although KPA categorizes pathways under another header. * One pathway associated also to NF-KB signaling.

Pathway associated with (total number of predicted pathways)	PRO (16)	Proline (2)	TEB (18)	Mix11 (10)	Folicur (6)	FLU (3)	Mix5 (1)	Mix6 (8)
Fatty acid, lipid, and cholesterol metabolism/homeostasis	–	–	–	2	–	–	–	–
TORC1 and TORC2 associated pathways	1	–	2	–	1	–		2
Other metabolism	1	–	–	–	–	–	–	1
ER stress	–		–	–	–	–	–	–
Chemotaxis/cell adhesion/cytoskeletal re-arrangements	–	–	2	–	–	–	–	–
Oxidative stress: ROS-induced cellular signaling	–	–	–	–	–	–	–	–
DNA damage	3	–	2	–	–	1	1	–
Other signal transduction	2	–	2	3	1	–	–	–
Number^of cytokine and i^mmune response^-associated pathways^	5	–	1	1	1	–	–	3 *

When analyzing transcript regulation based on the 196 GPS transcripts, Mix 7 induced differential abundance of 108 transcripts, Proline of 72, PRO of 113, and NND of 69 transcripts. Combining PRO and NND as in Mix 7 and in Proline led to the appearance of differentially abundant transcripts that were not identified in the cells treated with single substances (unique 31 of 108 for Mix 7, 6 of 72 for Proline). The most upregulated transcript in Proline- and Mix 7-treated cells was NACHT, LRR, and PYD domains-containing protein 12 (NLRP12). It had also higher expression levels in PRO- and NND-treated cells but exhibited the highest fold change, more than 4-fold, in Mix 7-treated cells. NRLP12 functions as a negative regulator of inflammation in myeloid cells [32].

### 3.7 Differentially expressed transcripts and/or proteins for case studies and KPA pathway analysis for PRO, TEB, NND, Mix 11, and Folicur Xpert

In this subgroup, the fungicides PRO and TEB were combined with NND to Mix 11 and Folicur Xpert. While TEB exposure led to 40 differentially abundant proteins, Mix 11 exposure resulted in only 7 proteins with different abundance, and the exposure to Folicur Xpert in 2 differentially abundant proteins. In Fig. S1, a multigroup comparison between control-treated cells, Folicur, Mix 11, NND, PRO, and TEB is visualized (FDR=0.05), leading to 72 differentially abundant proteins. Mix 11 and Folicur treatment induced a similar pattern (Fig. S1).

KPA analysis predicted 18 pathways affected by TEB exposure, summarized in Table 5. Several pathways were associated with autophagic processes and/or proteasomal activity. The top upregulated protein in response to TEB was Sequestosome-1 (p62/SQSTM1), a receptor necessary for selective macroautophagy [33]. Mix 11 and Folicur did not share many similarities based on KPA pathway prediction. However, input data for the KPA analysis uses a p-value cut-off of 0.05 instead of an FDR cut-off of 0.05 used for the identification of differentially abundant proteins. It is also worth noting that “Signal transduction_mTORC1 (Mammalian target of rapamycin) downstream signaling” is part of the predicted pathways in response to Folicur treatment, and though this is not labeled as “metabolism” by the software, regulation of TORC1 downstream signaling has a major impact on metabolic responses [34]. Pathway regulation predictions in response to Mix 11 and Folicur Xpert are summarized in Table 5.

Even more changes could be detected on the transcript level. 120 out of 196 transcripts were differentially abundant in response to TEB (similar level as PRO with 113 regulated transcripts, see above), also including Modulator of macroautophagy TMEM150B with an almost 5-fold change compared to controls. Mix 11 led to 102 differentially abundant transcripts compared to controls, whereof 20 were unique, i.e. had not appeared as differentially abundant in response to any other substance in this group. Among these 20 were several transcripts with roles in autophagy (Transmembrane protein 59 (TMEM59), Alkaline ceramidase 2 (ACER2), Tumor protein p53-inducible nuclear protein 1 (TP53INP1), Ras-related protein Rab-33B (RAB33B)); further ER degradation-enhancing alpha-mannosidase-like protein 2 (EDEM2), which may initiate the ERAD pathway [35] and others associated with signaling processes and metabolism. 70 transcripts were differentially expressed upon exposure to Folicur Xpert, whereof only 2 transcripts appeared as unique for the response to Folicur Xpert. Again, NLRP12 was one of the most abundant differentially expressed transcripts in response to PRO, TEB, NND, Mix 11, and Folicur Xpert, with the highest fold changes observed for TEB and Mix 11 treatment.

### 3.8 Differentially expressed transcripts and/or proteins for case studies and KPA pathway analysis for FLU, BEN, POL, Mix 4, Mix 5, Mix 6 and Shirlan

Few changes in protein abundances were detected after exposure to FLU (4 proteins), the most cytotoxic active ingredient investigated in this study. Moreover, mimicking the commercial formulation Shirlan by combining FLU and POL (Mix 5) and FLU, POL, and BEN (Mix 6) led to few changes, 1 and 2 proteins, respectively. Exposure to Shirlan did not result in any differentially abundant proteins compared to controls. In a heatmap (Fig. S2) based on a multigroup comparison (FDR < 0.05), the levels of the 55 differentially abundant proteins in response to Shirlan, FLU, BEN, POL, Mix 5 (FLU, POL), and Mix 6 (FLU, POL, BEN) are visualized. Mix 4 was not included in the proteomics analysis. FLU and the mix responses seem to result in a similar protein abundance pattern, while the patterns for the adjuvants BEN and POL clearly differ.

FLU exposure led to 40 differentially abundant transcripts. Shirlan induced changes in 2 transcripts, among them NQO1, which is also one of the three (NQO1, HMOX1, and TXNRD1) transcripts whose protein counterpart was found regulated on protein level (Suppl. Table 1, multigroup comparison, Fig. S2). The protein regulation pattern for BEN- and POL-treated cells sticks out in the heat map (Fig. S2).

Comparing the differentially abundant transcripts of each mix with those induced by each of its constituents alone, Mix 5 produces 4 unique transcripts, among them one associated with lipid homeostasis (1-acylglycerol-3-phosphate O-acyltransferase ABHD5 (ABHD5)) and one with autophagy (RUBCNL) [36], [37], while Mix 4 and Mix 6 result in no unique transcripts. Mix 5 also induced most differentially expressed transcripts, 28 in contrast to 18 for Mix 4 and 24 for Mix 6. NQO1, HMOX1, and TXNRD1 transcripts were found differentially abundant upon exposure to FLU, Mix 4, Mix 5, and Mix 6. While there is also a significant increase of NQO1 and TXNRD1 transcript levels in response to BEN treatment and of NQO1 in response to POL, FLU and Mix 6 show the highest and almost identical fold changes (Suppl. Table 1).

In summary, most single chemicals induced unique patterns of transcripts and protein regulation, including the adjuvants. Artificial mixes often induced more changes than associated PPPs. Proline was an exception with both many differentially abundant proteins and novel proteins not identified in cells treated with NND or PRO alone. However, input concentrations need to be taken into account here – PPPs may contain additional, for us not known ingredients, which affect the cytotoxicity profile and may e.g. lead to comparably lower input concentrations of the respective chemicals combined in the associated mixes. Generally, numerous novel entities appeared in the artificial mixes that were not detected as differentially abundant in the single chemical profiles. Cocktail effects seem to occur as judged by the increasing or decreasing magnitudes of responses, but also by the appearance of novel differentially expressed transcripts and/or proteins, which are exclusively detected after mixing different chemicals.

## 4 Discussion

In this study, we have investigated 3 fungicides, 3 adjuvants, and 3 associated fungicide formulations for their skin sensitizing properties in a DC cell-based model. Studies using *in vitro* methods for skin sensitization assessment in the context of agricultural chemicals have been published [38], [39], [40], [41], however, to our knowledge none with the chemicals investigated here or with focus on the mechanisms of potential cocktail effects. For some of the chemicals and PPPs, animal and/or human data indicating their skin sensitizing capacity/potency is available (Table 1). Existing classifications are mainly based on animal test data, and unfortunately, the comparison to available human data is sometimes neglected. We here provide predictions using the GARD® technology protocols, confirming the existing labels as skin sensitizers for the preservative BEN, the active principle FLU, and the formulations Folicur Xpert and Shirlan. Notably, the prevalence of contact allergy to BEN has risen during the past years, likely due to its increased use in household products [42].

Surfactants are included in many products and can enhance the skin sensitizing properties of accompanying chemicals in mixtures by impairing the skin barrier function [20], providing (additional) danger signals and contributing to inflammation [43], [44], [45]. In the context of skin sensitization, these danger signals could be stress- or damage-associated molecular patterns (DAMPs), such as released ATP and reactive oxygen species (ROS), as well as extracellular matrix components including fragmented hyaluronic acid, or the protein High-mobility group box 1 (HMGB1). These signals can lead to the release of different cytokines/chemokines by nearby (innate immune) cells creating a pro-inflammatory environment [16]. Notably, POL and NND are considered to be eye irritants. NND is further classified as a skin irritant and possible respiratory irritant (source substance infocards, European Chemical Agency, last accessed 27 April 2022).

We here discuss potential cocktail effects based on the above-described data with a focus on involved adjuvants. An important parameter to consider is the input concentration. For Mix 7 and 11, lower input concentrations than for single substances needed to be used to avoid excessive cytotoxicity. Also, for Mix 4, Mix 5 and Mix 6, the used concentrations of BEN and POL are much lower than for the single chemical treatments due to the high cytotoxicity of FLU and the subsequent need to go down further in BEN and/or POL concentration to retain the molar ratios when mimicking the associated fungicide formulation. Exposure of cells to Mix 11 (PRO+TEB+NND) resulted in increased CD86 expression (181%). Considering that the input concentration of TEB in Mix 11 was only half of the concentration tested for TEB alone and that PRO and NND alone did not trigger a significant change of CD86 expression, 181% compared to control would be consistent with a more than additive effect. The related commercial formulation Folicur Xpert did not seem to affect CD86 expression at all (Fig. 7). The respective inputs were comparable with 40 µg/mL Folicur Xpert and approximately 52 µg/mL combined input in Mix 11. Similarly, the input concentration for Mix 7 mimicking Proline (58 µg/mL) was around 53 µg/mL, and for Mix 6 mimicking Shirlan (12 µg/mL) approximately 12.5 g/mL. However, both Folicur Xpert and Shirlan contain additional components we could not obtain and use, which may influence the cytotoxicity profile. In addition, for all PPPs it is common that there are only concentration ranges given for several ingredients, making it impossible to properly mirror compositio

Mix 7 (PRO+NND) and Mix 11(PRO+TEB+NND) exposure led to differential expression of a similar number of transcripts (108 for Mix 7, 102 for Mix 11) and with 31 (Mix 7) and 20 (Mix 11) transcripts not appearing as regulated in response to their single constituents. The bulk of regulated transcripts, 52 transcripts, was shared between Mix 7-, NND-, and PRO-treated cells, and similarly, 46 transcripts were shared by Mix 11-, PRO-, TEB-, and NND-treated cells. Of these, 44 overlapped. When comparing the responses to Mix 7 and Mix 11 exposure directly with each other, the majority, 90 differentially expressed transcripts, are common, also including the autophagy-associated transcripts TMEM150B, ACER2, TP53INP1, and RAB33B. The remaining transcripts are unique for each mix, which indicates that all chemicals contribute, possibly proportional to their concentration. NND is used at a very similar concentration in both, but PRO in a 3x higher concentration in Mix 7. A similar number of transcripts were exclusively shared between NND and respective mix, while 20 transcripts were exclusively shared with PRO and Mix 7, and only 6 between PRO and Mix 11. However, the interplay is complex, already for binary mixtures of chemicals.

FLU is classified both by CLP and by us as category 1A, a strong skin sensitizer [46]. It clearly dominated the cytotoxicity profile for Mix 4, Mix 5, and Mix 6. However, FLU induced few changes on protein level with 4 differently expressed proteins: MX1, WD repeat-containing protein 43 WDR43, Bifunctional glutamate/proline-tRNA ligase (EPRS), and mitochondrial Pyrroline-5-carboxylate reductase 1 (PYCR1) (Suppl. Table 1). Two of these are induced by (MX1) or are responsive to IFN-γ (EPRS). EPRS is further reported to be an effector of the mTORC1 signaling pathway, playing a role in fat metabolism in a murine study [47]. PYCR1 is involved in proline biosynthesis and is suggested to participate in the cellular oxidative stress response [48]. Notably, the amino acid proline itself is considered to protect mammalian cells against oxidative stress [49]. These tentative responses are mirrored by the transcripts with the highest fold changes in response to FLU. Higher abundance transcripts are e.g., HMOX1, NQO1, TXRND1, consistent with a role of oxidative stress in skin sensitization [16], [50], further lysophosphatidic acid receptor 1, LPAR1. LPAR1 is critical for the re-organization of the actin cytoskeleton and migration but it also modulates lipopolysaccharide-induced inflammation in mice [51], [52]. Less abundant compared to controls are transcripts connected to cholesterol homeostasis: NADPH:adrenodoxin oxidoreductase (FDXR), the drug-binding orphan receptor TMEM97 [53], and high mobility group protein B3 (HMGB3), resembling the DAMP HMGB1. HMGB1 may play a central role in the development of ACD and other inflammatory diseases [54].

To mimic Shirlan, FLU was combined with BEN and/or POL in the ratios present in Shirlan resulting in Mix 4–6 (Table 1). NQO1, HMOX1, and TXNRD1 transcripts were found at higher levels after FLU, Mix 4, Mix 5, and Mix 6 exposure, for NQO1 and HMOX1, indicating the induction of oxidative stress. Judged by the fold changes, FLU seemed to dominate these responses to the mixes (Suppl. Table 1).

Mix 5 (FLU and POL) exposure led to most newly appearing transcripts compared to the transcripts differentially expressed in response to its constituents (4 of 28 transcripts, ABHD5, RUBCNL, RNA pseudouridylate synthase domain-containing protein 2 (RPUSD2), Ribosome Production Factor 2 Homolog Pseudogene 1 (RPF2P1)). Interestingly, the autophagy-related transcript, Pacer (RUBCNL/KIAA0226L) appears to be involved in the signal integration in the late stages of autophagy and lipid metabolism downstream of master regulator mTORC1 [36], [37], linking to the changes in lipid and cholesterol homeostasis/biosynthesis induced by POL exposure.

Interestingly, the main modes of action of pesticide immunotoxicity include oxidative stress, mitochondrial dysfunction, ER stress, disruption of the ubiquitin-proteasome system, and autophagy impairing immune cell function [5], which is exactly what we found in the DC model used here. Future work should focus on validating the here discussed pathways and processes including several time points and dose-response curves with approaches such as RT-PCR, Western blot, etc. Since we only investigate protein and transcript expression after 24 h of incubation, we may in the current study miss candidates for which responses occur early or delayed.

The metabolism of immune cells, including DCs’, has recently received much attention due to the insight that different phases of immune cell activation are tightly linked to cellular metabolism to meet the current bioenergetic and -synthetic needs of the cells [55]. mTOR, a Ser/Thr protein kinase with nutrient sensor function, is an important regulator in the DC metabolic changes, including lipid and cholesterol metabolism, in DCs related to their function and immune responses [56]. Lühr et al. reported that the overall lipid composition was changing significantly during primary human monocyte-derived DC (moDC) maturation, rendering mature moDCs stiffer than their immature counterpart. These changes play an important role during cell migration and T cell activation [57].

Much of the described work has focused on microbial stimuli. However, we also saw indications in a previous -omics study [39] using the same DC model as in this study that cholesterol homeostasis and biosynthesis seemed to be affected in response to a surfactant used in PPPs. Recently, a study compared the DC response of human moDCs to LPS and the skin sensitizer NiSO_4_ [58] and found that the exposure to NiSO_4_ induced cholesterol depletion, which the cells counteracted by inducing genes and proteins connected to cholesterol biosynthesis. Thus, it seems highly motivated to further investigate the role of cholesterol regulation in DCs in response to xenobiotics.

In addition, autophagy, “self-eating” has been shown to play an important role in DC function. The process comprises the ingestion, degradation, and recycling of components and it can regulate different aspects of innate and adaptive immunity and inﬂammation as reviewed in [59], [60]. There is a clear connection between autophagy, metabolism, and mTOR signaling as one of the three systems to regulate autophagy is controlled by AMP-activated protein kinase, a master regulator of metabolism and mTOR [59], [61]. Interestingly, pathogen-associated molecular patterns (PAMPs) and DAMPs can serve as “Signal 0″, i.e. induce autophagy before subsequent steps take place in the upcoming immune response [62]. Autophagy is also described to act both up- and downstream of toll-like receptor (TLR) signaling [60]. We and others have previously seen indications of autophagy regulation in response to skin sensitizers [39], [63] and we again see transcripts and proteins linked to autophagy in response to e.g., the fungicide TEB in this study. Autophagic adaptors, such as SQSTM1/p62-like receptors (“SLRs"), have been suggested as pattern recognition receptors [59], [64].

The role of redox networks is not only accepted in skin sensitization, but they also seem to integrate signaling pathways in cells more in general. Levonen *et al.* describe cysteine-containing, redox-sensing proteins as an “electrophile-responsive proteome”, which is connected to pathways responding to oxidative stress: the KEAP1-NFR2 pathway, the heat shock response, the unfolded protein response (UPR), redox regulation of autophagy and vice versa, and further the integration of metabolism and cellular energetics with redox signaling [65]. The relevance of UPR for skin sensitization is also highlighted by a recent study where UPR activation or inhibition in response to sensitizers with different potencies and associated inflammatory responses *in vitro* and *in vivo* are investigated. The authors report synergistic UPR activation and NF-κB translocation when combining weak sensitizers with SDS [66]. Another study has addressed the relationship between skin sensitizers and the induction of oxidative and ER stress during the maturation of DC-like cells and reported that skin sensitizer 1-ﬂuoro-2,4-dinitrobenzene induced ROS-dependent activation of the ERK–eIFα–ATF4 UPR while it also upregulated autophagy-related genes in THP-1 cells [63].

In summary, we see complex responses visible both on transcript and protein levels even with low concentrations of chemicals, including adjuvants, and new entities and pathways appearing when combining several chemicals, which supports the occurrence of cocktail effects. Further work would be needed to validate the here presented findings, such as experiments in other cell models, but our data clearly supports that all chemicals in a PPP can affect toxicity, and thus should be considered in the risk assessment. The number of differentially expressed molecules is not necessarily decisive, it also depends on what molecules and pathways are influenced. A chemical or a mixture can be, according to our data, strongly skin sensitizing (FLU) despite few changes on protein level, while others (NND) induced clear responses both on protein and transcript level despite showing no/weak skin sensitizing capacity according to CLP and our *in vitro* data. Notably, NND exposure also reduced IL-8 secretion compared to untreated controls, and all investigated mixes containing it were predicted as weak sensitizers even if there were other chemicals present classified as strong sensitizers.

In conclusion, improving our knowledge about the role of inflammation for immune responses involving DCs could aid in understanding the variations in sensitization potency shown by contact allergens, and, as a consequence, also improve skin sensitization risk assessment [67]. We believe, the same holds true to better understand and predict cocktail effects, and that the concept and insights may as well serve for a better understanding and improved prediction of immunotoxic effects in general.

## CRediT authorship contribution statement

**Renato de Àvila:** Conceptualization, Writing – original draft, Writing – review & editing, Visualization, Investigation, Formal analysis, Data curation. **Sofía Carreira Santos:** Investigation, Writing – review & editing. **Valentina Siino:** Investigation, Writing – original draft, Writing – review & editing. **Fredrik Levander**: Methodology, Formal analysis, Data curation, Writing – review & editing. **Malin Lindstedt:** Funding acquisition, Methodology, Writing – review & editing. **Kathrin Zeller**: Conceptualization, Funding acquisition, Methodology, Formal analysis, Data curation, Writing – original draft, Writing – review & editing, Investigation, Visualization, Project administration.

## Declaration of Competing Interest

The authors declare that they have no known competing financial interests or personal relationships that could have appeared to influence the work reported in this paper.

## Data Availability

Associated data is deposited and/or can be provided upon request.

## Footnotes

Appendix A Supplementary data associated with this article can be found in the online version at doi:10.1016/j.toxrep.2022.11.004.
