# Supplementary material for: Adjuvants in fungicide formulations can be skin sensitizers and cause different types of cell stress responses
Source: Toxicol Rep. 2022 Nov 18;9:2030–41. doi: 10.1016/j.toxrep.2022.11.004 (PMC9742974; doi:10.1016/j.toxrep.2022.11.004)
Supplement: Supplementary file 1 — Supplementary material. [file mmc1.docx]

**Supplementary data de Ávila et al., 2022**


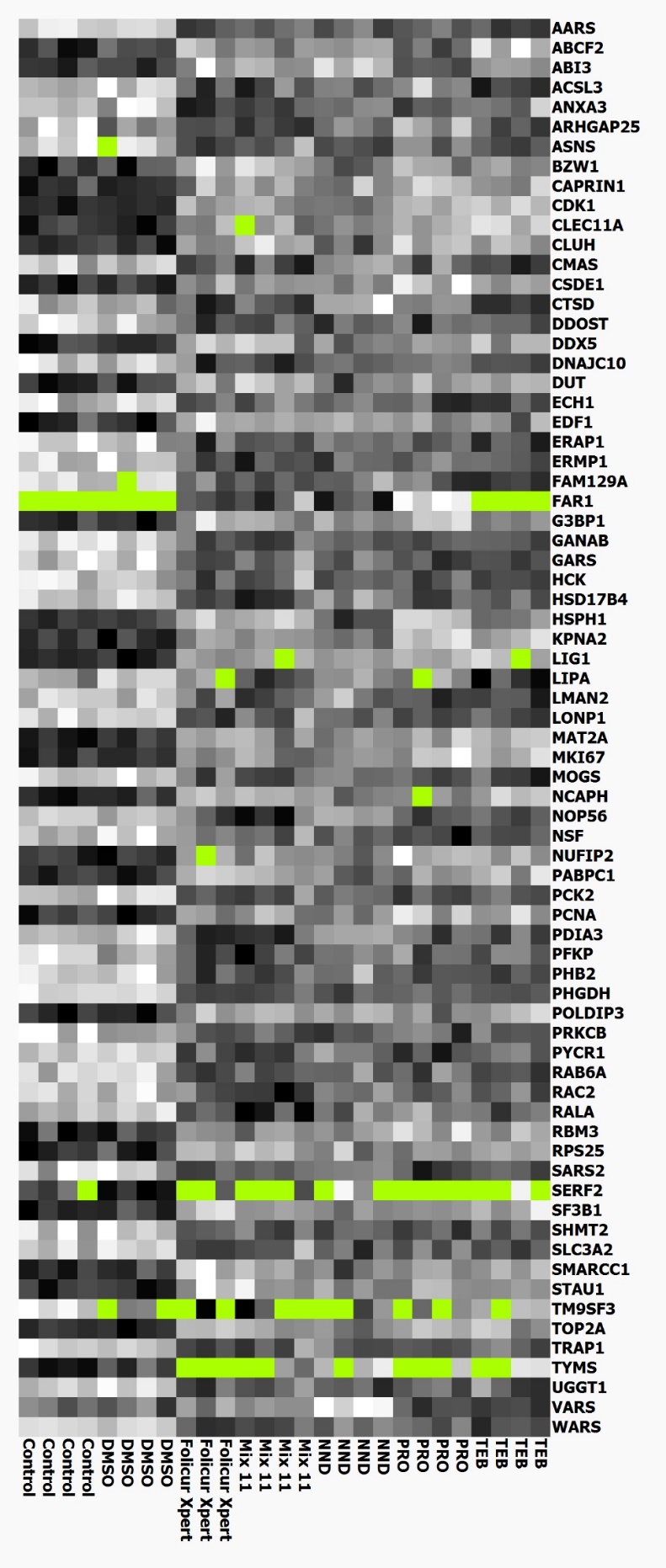
**Figure S1.** (Color) **Heatmap illustrating the multigroup comparison (FDR < 0.05, 72 differentially expressed proteins) between the proteomes of cells exposed to indicated treatments.** Dark color indicates high expression levels, white low. Missing values are shown in light green, normalized to mean=0, variance=1.


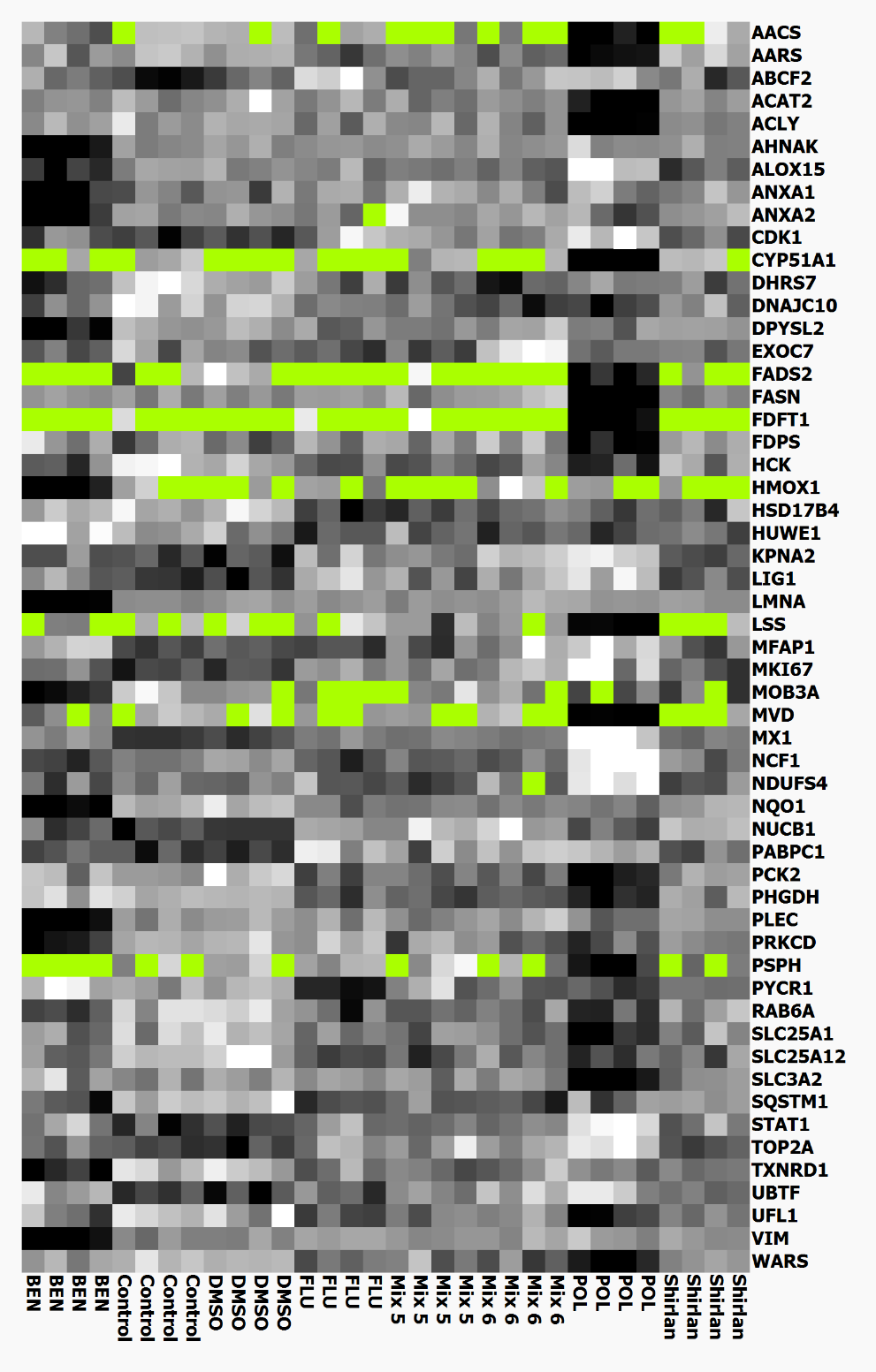


**Figure S2.** (Color) **Heatmap illustrating the multigroup comparison (FDR < 0.05, 50 differentially expressed proteins) between the proteomes of cells exposed to indicated treatments.** Dark color indicates high expression levels, white low. Missing values are shown in light green, normalized to mean=0, variance=1.
